# Homeobox code model of heterodont tooth in mammals revised

**DOI:** 10.1038/s41598-019-49116-x

**Published:** 2019-09-06

**Authors:** Yoshio Wakamatsu, Shiro Egawa, Yukari Terashita, Hiroshi Kawasaki, Koji Tamura, Kunihiro Suzuki

**Affiliations:** 1 Department of Developmental Neuroscience, United Centers for Advanced Research and Translational Medicine (ART), Tohoku University Graduate School of Medicine, Sendai, Miyagi 980-8575 Japan; 2 Department of Ecological Developmental Adaptability Life Sciences, Tohoku University Graduate School of Life Sciences, Sendai, 980-8578 Japan; 3 Department of Medical Neuroscience, Kanazawa University, Graduate School of Medicine, Kanazawa, 920-8640 Japan; 4 Research Institute of Oral Science, Nihon University School of Dentistry at Matsudo, Chiba, 271-8587 Japan

**Keywords:** Ectoderm, Evolutionary developmental biology

## Abstract

Heterodonty is one of the hallmarks of mammals. It has been suggested that, homeobox genes, differentially expressed in the ectomesenchyme of the jaw primordium along the distal-proximal axis, would determine the tooth classes (homeobox code model) based on mouse studies. Because mouse has highly specialized tooth pattern lacking canine and premolars (dental formula: 1003/1003, for upper and lower jaws, respectively), it is unclear if the suggested model could be applied for mammals with all tooth classes, including human. We thus compared the homeobox code gene expressions in various mammals, such as opossum (5134/4134), ferret (3131/3132), as well as mouse. We found that *Msx1* and *BarX1* expression domains in the jaw primordium of the opossum and ferret embryos show a large overlap, but such overlap is small in mouse. Detailed analyses of gene expressions and subsequent morphogenesis of tooth germ in the opossum indicated that the *Msx1*/*BarX1* double-positive domain will correspond to the premolar region, and *Alx3*-negative/*Msx1*-positive/*BarX1*-negative domain will correspond to canine. This study therefore provides a significant update of the homeobox code model in the mammalian heterodonty. We also show that the modulation of FGF-mediated *Msx1* activation contributes to the variation in the proximal *Msx1* expression among species.

## Introduction

Heterodonty is one of the hallmarks of mammalian lineage, and it has been gradually achieved during the course of synapsid evolution^1^. The current mammalian tooth classes include incisor, canine, premolar, and molar, from mesial tip to temporal end of the jaw (in this study, such directionality is referred to as “distal-proximal axis”), and all the tooth classes had already emerged in Mesozoic mammaliaformes/mammals^1^. Each tooth classes can be used for specialized functions, supporting the prosperity of mammals in the modern world. Further modifications of the tooth pattern might have also contributed to the ecomorphological specialization and the niche partitioning of mammals^1^.

Previous studies have suggested that the regionalized expression of the homeobox transcription factors in the neural crest-derived ectomesenchyme along the distal-proximal axis of the jaw primordium is involved in the tooth class determination^2–4^. McCollum and Sharpe subsequently advocated the “homeobox code model” of dental patterning, suggesting that, along the distal-proximal axis of the jaw primordium, *Alx3*/*Msx1*-double-positive domain, *Alx3*-negative/*Msx1*-positive domain, and *Msx1*-negative/*BarX1*-positive domain would give rise to incisors, canine + premolars, and molars, respectively^3,4^. Such regionalized expression domains of homeobox genes are established, at least in part, by the epithelium-derived growth factors, such as *FGF8* and *BMP4*^2,5–9^. In subsequent development, tooth initiation and morphogenesis require extensive reciprocal interactions between epithelium and mesenchyme mediated by signal transduction pathways, such as Bmp, Fgf, Wnt, and Hedgehog^10–12^. How the morphology of each tooth class is established is largely unknown, but the number of enamel knots will determine the number of cusps, as enamel knots act as signaling centers to organize the morphogenesis by regulating cell proliferation and movement^13–15^. Although underlying mechanism(s) that link(s) the regional homeobox expression in the jaw primordia and later tooth morphogenesis is largely unknown, a previous study of *BarX1* expression in the mesenchyme of multi-cusp tooth primordia^16^ indicates the initial code might be inherited in the tooth germ mesenchyme.

The homeobox code model had been proposed largely based on the data obtained from mouse studies, despite the fact that the mouse has a highly specialized dentition, possessing only 1 incisor and 3 molars for each quadrant of the jaw, separated by a diastema, a tooth-less region. Thus, it was unclear if this model could be directly applicable to other mammals, particularly to the species with all prototypical tooth types such as human. There are a few studies describing tooth development in species with all tooth types, such as ferret (*Mustela putorius furo*)^17–19^, house shrew (*Suncus murinus*)^20,21^, and gray short-tailed opossum (*Monodelphis domestica*)^22^. There are also some studies examining the expression of homeobox genes in various mammalian species^23,24^, but close comparisons of the homeobox code in mammalian species has not been done.

In this study, we have compared the expression of homeobox code transcription factors in the jaw primordium of various mammalian species, including mouse, ferret, and opossum, and found a large *Msx1*/*BarX1*-double-positive domain in the middle of the jaw primordium of the ferret and opossum embryos. As previously revealed, in contrast, such domain is small in the mouse mandibular arch. Histological analyses on various developmental stages of opossum suggest that the *Msx1*/*BarX1*-double-positive domain may correspond to premolars. Close observations of the homeobox expressions in both upper and lower jaws indicate that the canine would correspond to *Alx3*-negative/*Msx1*-positive/*BarX1*-negative region. Our explant culture studies indicate that the proximal expression of *Msx1* in the mandibular arch is activated by FGF signaling. Thus, we suggest a revision of the homeobox code model in mammalian dentition. We also suggest that the diastema in mouse upper and lower jaws might be achieved, in part, by distinct mechanisms.

## Materials and Methods

### Experimental animals

Animal experiments were conducted in accordance with the guidelines of Tohoku University (Regulations for Animal Experiments and Related Activities at Tohoku University), with approval of the Tohoku University Medical School Animal Experiment Committee (2015MdA-129-1, 2017MdA-209, 2018MdA-063).

Japanese quail (*Coturnix japonica*) eggs were obtained from local farms (Quail Factory-Tydess). Pregnant B6 mice were purchased from CLEA Japan, and midday of the vaginal plug was designated as embryonic day 0.5 (E0.5). The breeding colony of gray short-tailed opossum, is maintained at Nihon University School of Dentistry at Matsudo, under the approval of Nihon University Animal Care and Use Committee (No. AP12MD015, AP14MD014). Opossum embryos were obtained from pregnant females, with mating video-recorded and visually confirmed. Ferrets were maintained at Kanazawa University under the approval of Kanazawa University Animal Care and Use Committee as described previously^25,26^. Ferret embryos were obtained from pregnant female ferrets. The Committee for Animal Experiment of Tohoku University Graduate School of Medicine approved the experimental procedures in this study.

### *In situ* hybridization

Whole-mount and section *in situ* hybridizations were performed as described previously^27^. Embryos were often bisected at the midline, and hybridized with distinct probes to accurately compare the expression of two genes at the identical developmental stage. The DNA fragments of opossum *Alx3*, *Msx1*, *BarX1*, *Dlx1/2*, and *FGF8*, corresponding to the exons were PCR-amplified from either oligo (dT) primed neonatal brain cDNA pool or genomic DNA prepared from *M*. *domestica* adult liver^28^. DNA fragments of ferret *Msx1* and *BarX1* were PCR-amplified from genomic DNA. The amplified DNA fragments were subcloned into *pBluescriptII* (Stratagene). The sequences of primers were listed in Supplementary Table 1. The opossum *BMP4*^29^, chick *Alx4*^30^, chick *Msx1*^31^, and chick *BMP4*^31^ have been described previously. cDNA clones of chick *BarX1*^32^, chick *FGF8*^33^, chick *Dlx1/2*^34^, mouse *Alx3*, mouse *BarX1*^2^, mouse *BMP4*, and mouse *FGF8*^35^ were kind gifts from Drs. Yasuyo Shigetani, Sumihare Noji, Isabell Miletich, Andrew Groves, Gen Yamada, Shinji Takada, and Tatsuya Sato. Mouse *Dlx2* cDNA was commercially obtained (Dnaform, Phantom Clone AK144652).

### 3D reconstruction

Postnatal opossums were decapitated and heads were fixed in 4% PFA/PBS. Brain and skin were removed, and the remaining jaw elements were embedded in paraffin. Serial sections were stained with hematoxylin and eosin with a standard method, and were photographed with KYENCE BZ-X710 microscope system. Tooth germs in the obtained images were manually filled, and the images were processed with Amira 5. 4. 3 (VSG Systems) for 3D reconstruction.

### Explant culture

Explant culture of the mandibular arch and transplantation of drug-soaked bead were performed mostly as previously described^8^. Mandibular arch along with flanking tissues were dissected from E11.0 *M*. *domestica*, E10.0 mouse, and E3 quail embryos. One AG1-X2 (formate form) anion exchange bead (Bio Rad), soaked with either 10 mM SU5402 (197-16731, WAKO) in DMSO or DMSO alone, was transplanted into each mandibular arch. Explants were placed onto Millicell-CM culture plate insert (0.4um pore size, Millipore), and cultured in DMEM medium (Gibco) for 24 hours. Explants were subsequently fixed in 4% PFA/PBS, and processed for whole-mount *in situ* hybridization, as indicated above.

### Correlation analysis of positions of tooth and mental foramen

Various mammalian skulls were digitally photographed and examined to correlate the position of mental foramen relative to the tooth along the proximal-distal axis of the dentary at Nihon University School of Dentistry at Matsudo (Chiba, Japan), the National Museum of Nature and Science (Tokyo, Japan), and the Ibaraki Nature Museum (Ibaraki, Japan). Specific specimen numbers are described in the figure legends and Supplementary Table 2.

## Results

### Homeobox gene expression and tooth classes in opossum jaw development

**Figure 1:**
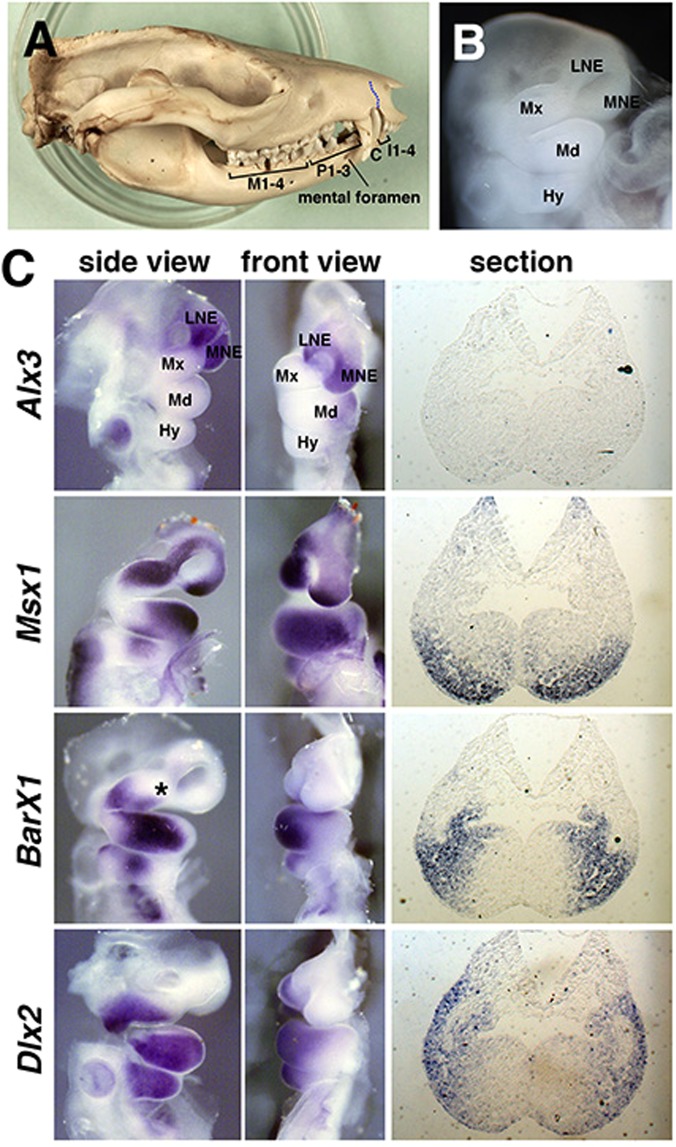
Dental formula of gray short tailed opossum and expression of homeobox code transcription factors in jaw primordium of E11.5 embryos. (**A**) Adult skull of opossum (KQN-m-011), showing dental formula. I1-4, C, P1-3, and M1-4 indicate incisors, canine, premolars, and molars, respectively. Note the position of mental foramen at the level between first and second premolars along the distal-proximal axis of the mandible. Blue dotted line indicates premaxilla-maxilla boundary. (**B**) A side view of the head region of E11.5 opossum embryo, showing jaw primordium. Hy: hyoid arch, LNE: lateral nasal eminence, Md: mandibular arch, MNE: medial nasal eminence, Mx: maxillary process. (**C**) Expression of homeobox code transcription factors in the jaw primordium of E11.5 embryos (left panels: whole-mount side views, middle panels: whole-mount frontal views, right panels: serial sections). See large overlap of *Msx1* and *BarX1* both in maxillary process and mandibular arch. Asterisk indicates *Alx3*-negative, *Msx1*-positive, *BarX1*-negative domain in the maxillary process.

As opossum has all tooth classes (dental formula: 5134/4134, Fig. 1A), we considered it as a potential model to uncover the prototypical state of the “homeobox code” in mammalian dentition. Thus, we first examined the expression of the homeobox code transcription factors in the jaw primordium of opossum (Fig. 1C,D). At 11.5 days post-coitum (E11.5, Fig. 1B), when distal tips of left and right mandibular arches meet at the midline, distal-proximal-ordered expression of the “homeobox code” genes, such as *Alx3*, *Msx1*, *BarX1*, and *Dlx2*, was examined both in mandibular and maxillary primordium on whole-mount preparations (Fig. 1C). This pattern was largely similar to that in mouse mandibular arch at comparable stages, such as E10.5, as previously described^3,4^ (See also Supplementary Fig. 1). Interestingly, however, we have observed a large overlap of the proximal part of the *Msx1* domain and most of the *BarX1* domain in the opossum mandibular arch (Fig. 1C). This *Msx1*-*BarX1* overlap was further confirmed on neighboring sections (Fig. 1C). In contrast, there is only a small overlap of expression domain of *Msx1* and *BarX1* in mouse mandibular arch^3,4^ (See also Supplementary Fig. 1).

**Figure 2:**
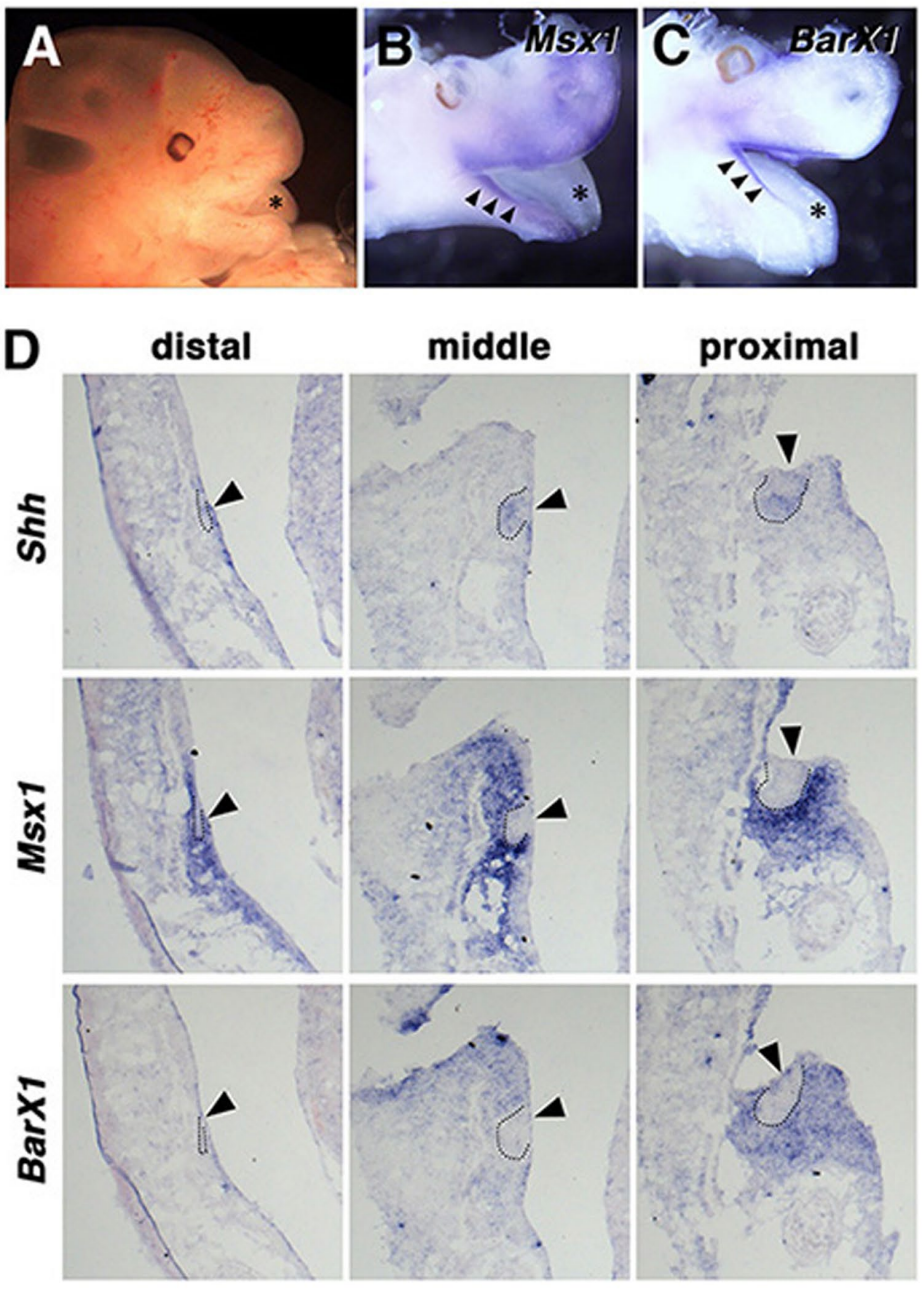
Expression of *Msx1* and *BarX1* in E13.5 and P0 opossum. (**A**) A side view of the head region of E13.5 opossum embryo. Asterisk indicates tongue. (**B**,**C**) Expression of *Msx1* and *BarX1* in E13.5 embryo. Brain was removed from an embryo, the remaining head was cut at the midline, and the right and left halves were hybridized either with *Msx1* or *BarX1* probe. For a better comparison, the picture of left half is horizontally flipped (**C**). See a large overlap of *Msx1* and *BarX1* both in the upper and lower jaws (arrowheads). Asterisks indicate tongue. (**D**) Expression of *Msx1* and *BarX1* in P0 opossum. To examine distal tooth germs, longitudinal sections were prepared. To examine middle and proximal tooth germs, frontal sections were prepared. *Shh* expression indicates the epithelial component of the tooth germs (arrowheads). While *Msx1* is expressed in the ectomesenchyme of tooth germs at all axial levels examined, *BarX1* expression is restricted to the middle and proximal mesenchyme. Dotted lines indicate epithelial buds.

We hypothesized that the difference of the “homeobox code” gene expression between the mouse and opossum mandibular arches described above might reflect the difference in the dental formula of these species. Because lineage-tracing method of mandibular arch cells at distinct distal-proximal axis in the opossum embryos is currently not available, we cannot directly assess the correlation of the homeobox-coded domains and the fate of the mandibular cells in distinct domains. As the *BarX1* expression in the proximal part of embryonic jaw primordia persists at later developmental stages in mouse^16,36^, we examined the expression of *Msx1* and *BarX1* at later stages of development to gain perspective on the spatiotemporal expression of *Msx1* and *BarX1* in the tooth germ. At E12.5 and E13.5 (Fig. 2A–C and Supplemental Fig. 2), a large overlap of the *Msx1* and *BarX1* was still observed on whole-mount preparations (Fig. 2B,C and Supplemental Fig. 2). At E14.5 (post-natal day 0, P0), continuous dental lamina/tooth bands along the distal-proximal axis were observed both in upper and lower jaws, and the *Shh*-positive tooth epithelium is mostly at the bud stage (Fig. 2D). The most distal tooth germ mesenchyme expressed both *Alx3* (not shown) and *Msx1*, but not *BarX1* (Fig. 2D). In more proximal part of the tooth band, tooth germ mesenchyme expressed both *Msx1* and *BarX1* (Fig. 2D). Thus, although the later expression of *Msx1* might be the result of local epithelial-mesenchymal interactions within the tooth germs^10–12^, the overlapped expression of *Msx1* and *BarX1* in the jaw primordium appeared to be maintained at least from E11.5 to P0 of opossum (Figs 1 and 2).

**Figure 3:**
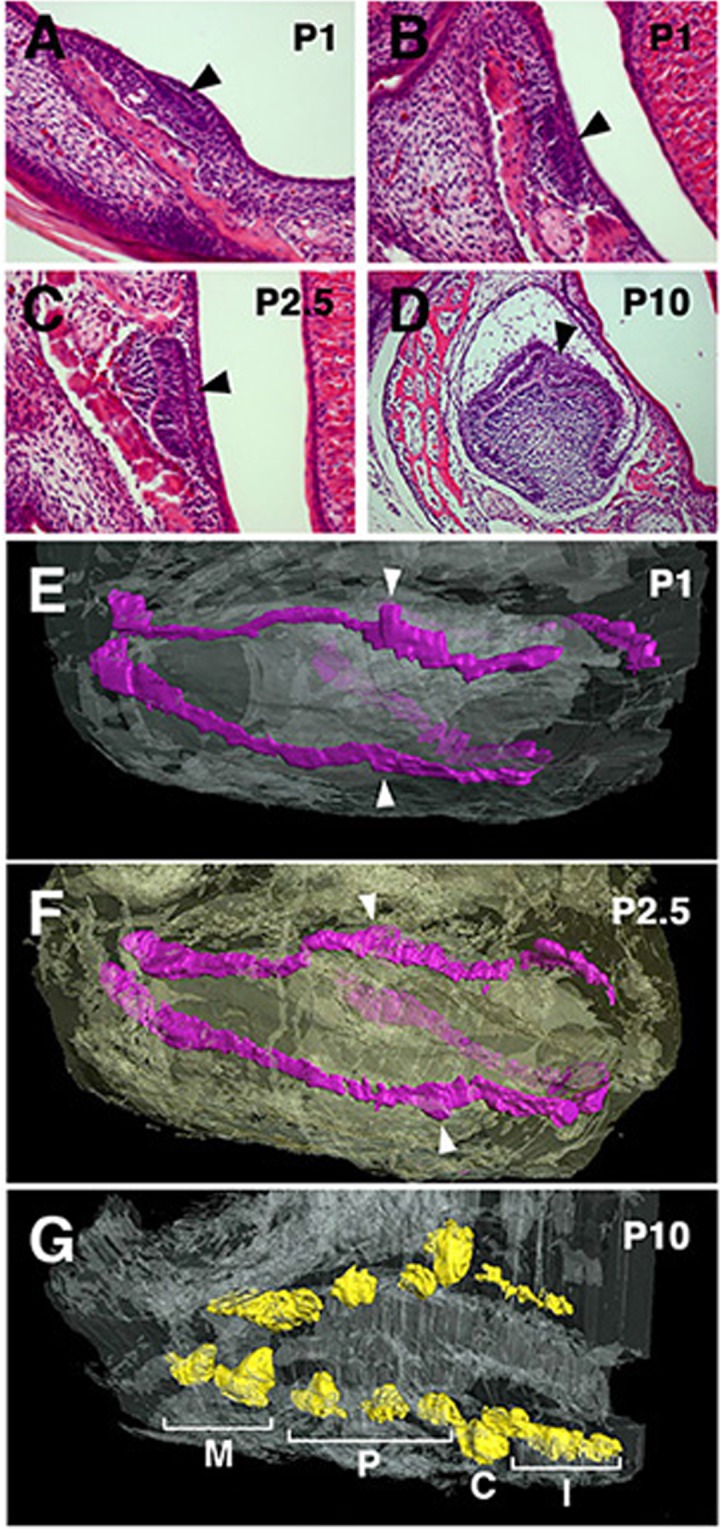
Morphology of the tooth germs in P1, P2.5, and P10 opossum infants. (**A**–**D**) Examples of hematoxylin-eosin-stained sections used for the 3D reconstructions of tooth germs in (**E**–**G**). Bud (**A**), early cap (**B**), late cap (**C**), and bell (**D**) stages of tooth germs are shown (arrowheads). (**E**.**F**) 3D reconstruction of tooth bands at P1 and P2.5. Tall tooth germs (arrowheads) in each tooth bands likely correspond to the future canines. (**G**) 3D reconstruction of tooth germs at P10. I, C, P, and M indicate incisors, canine, premolars, and molars, respectively.

To correlate the tooth types and the expression domains of homeobox code genes, particularly *Msx1* and *BarX1*, we further examined the morphology of the tooth band and tooth germs at later stages, such as P1, P2.5, and P10. For this purpose, we have made serial sections (Fig. 3A–D), to generate 3D images of the tooth germs (Fig. 3E–G, see also Supplemental Movie 1–3). At P1, we could observe sub-regionalization of the tooth band. Around the distal one third of the tooth band, the tooth germ epithelium at the bud stage appeared significantly taller (Fig. 3E, Supplementary Movie 1). The tooth germs located distally and proximally to this tall epithelium, were apparently smaller, but at the most proximal part of the tooth band, tooth germs were bigger at the early cap stage (Fig. 3B,E). This size tendency appeared intact at P2.5, although most tooth germs are at the late cap stage (Fig. 3C,F, Supplementary Movie 2). At P10, individual tooth germs were at the bell stage, and are morphologically distinguishable (Fig. 3D). Incisor region and premolar-molar region were clearly separated by canine (Fig. 3G, Supplementary Movie 3). Based on the position and morphology during the course of the development, the tallest tooth germ at P1 and P2.5 would most likely correspond to the canine. Considering the broad overlap of *Msx1* and *BarX1* expression domains in the mandibular arch, the proximal *Msx1*/*BarX1*-double-positive region likely corresponds to the premolar region in the opossum lower jaw, while the distal *Msx1*-positive/*BarX1*-negative domain will correspond to the incisor region (Fig. 5. See the next paragraph for the homeobox code for the canine).

**Figure 4:**
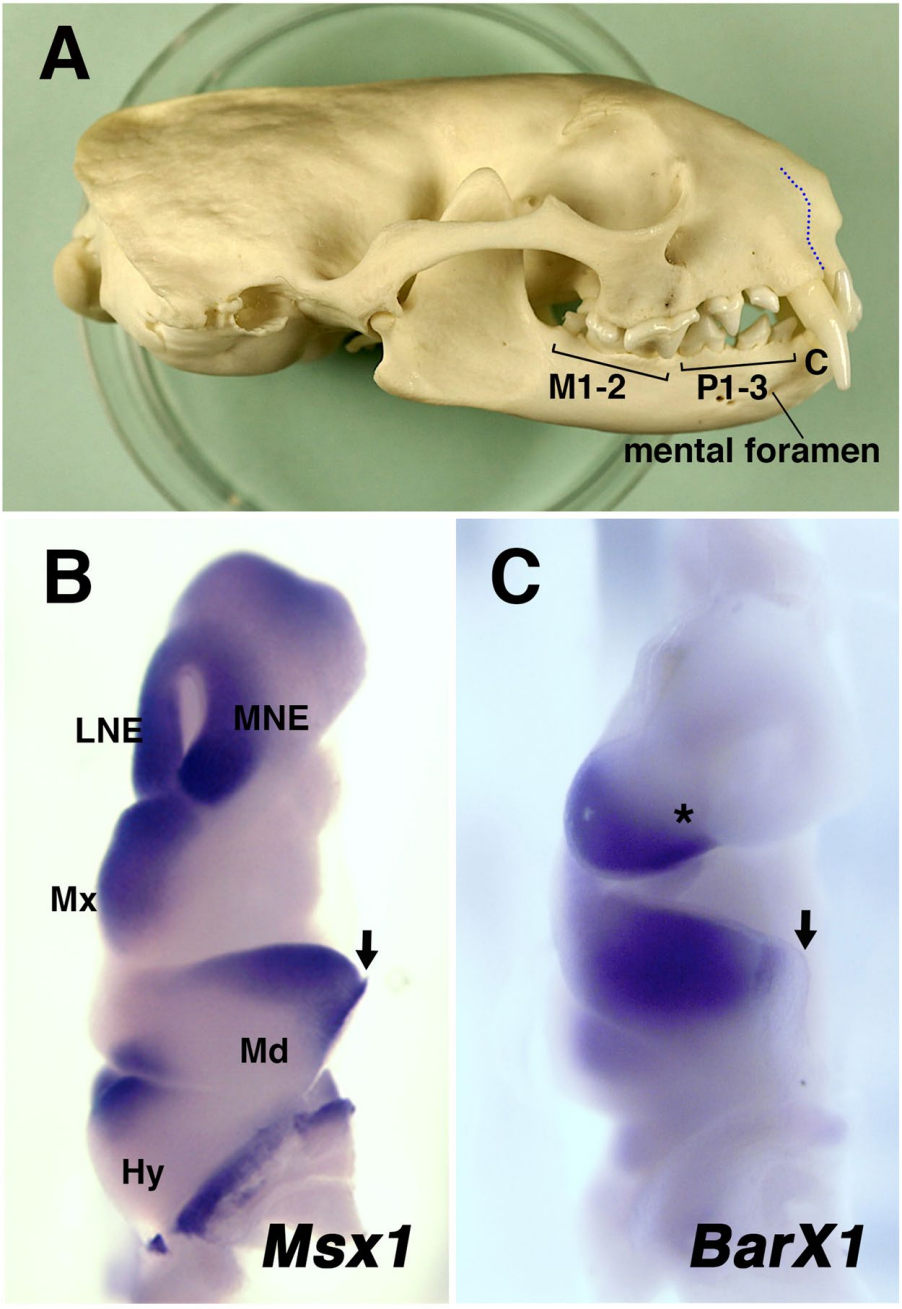
Dental formula of ferret. and expression of *BarX1* and *Msx1* in jaw primordium of E20 embryos. (**A**) A side view of adult ferret skull (SUG-m-001), showing dental formula. C, P1-3, and M1-2 indicate canine, premolars, and molars, respectively. Incisors are not visible in this picture. Blue dotted line indicates premaxilla-maxilla boundary. Note the position of mental foramen at the level between first and second premolars of the mandible. (**B**) A front view of the head region of an E20 ferret embryo, showing *Msx1* expression in the jaw primordium. An arrow indicates the distal tip of the mandibular arch. Hy: hyoid arch, LNE: lateral nasal eminence, Md: mandibular arch, MNE: medial nasal eminence, Mx: maxillary process. (**C**) Expression of *BarX1* in an E20 ferret embryo. Distal limit of the expression is very close to the tip of the mandibular arch (arrow). Large overlaps of *Msx1* and *BarX1* domains are found both in maxillary process and mandibular arch. Asterisk indicates *Msx1*-positive, *BarX1*-negative domain in the maxillary process.

**Figure 5:**
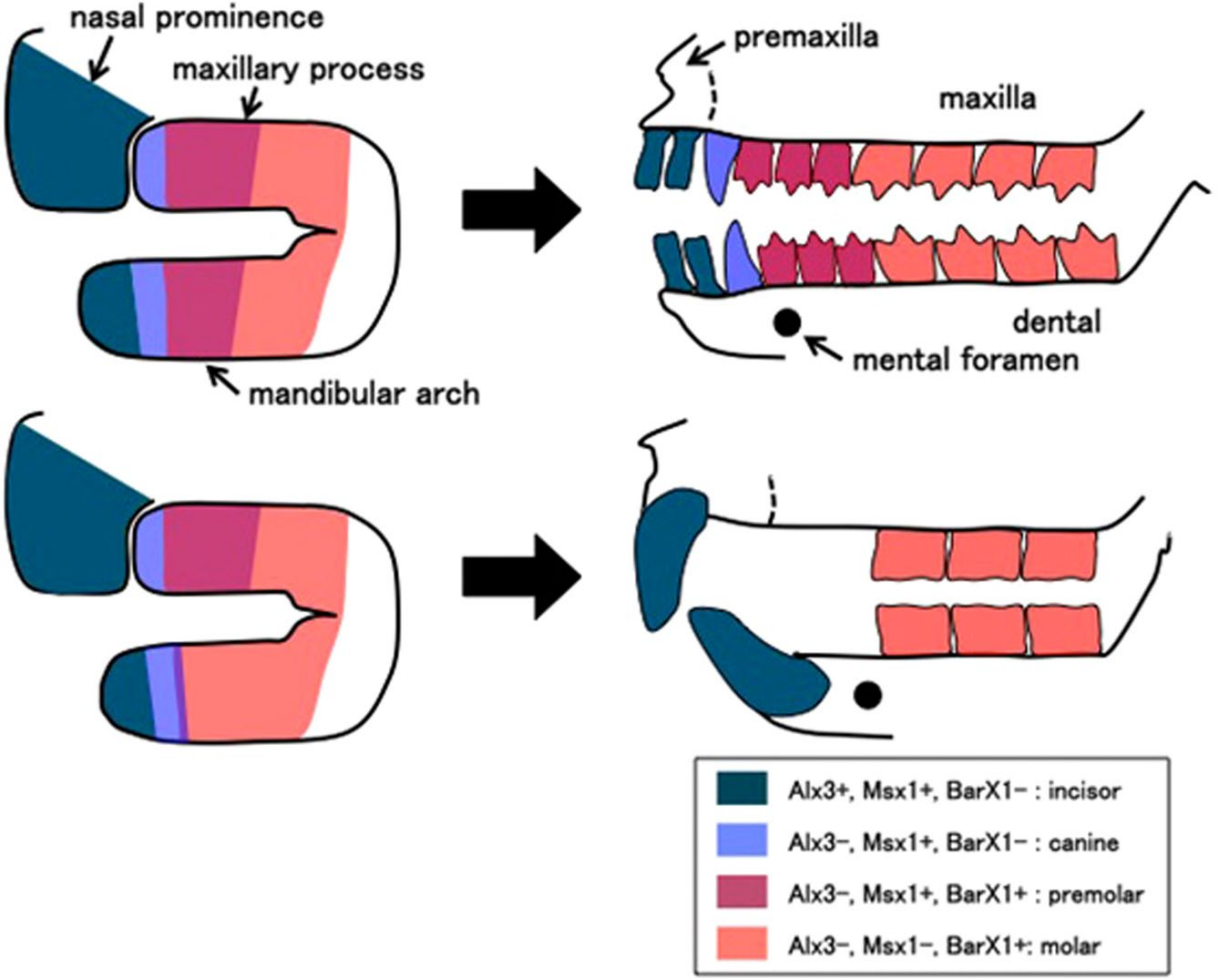
Correlation of the homeobox code expression and tooth classes in the prototypical mammal (upper) and rodents (lower). Upper figure shows the fourth molar, as placentals (eutherians) and marsupials (metatherians) have 3 and 4 molars, respectively.

The mandibular arch has no apparent morphological landmark along the proximal-distal axis to correlate the homeobox expressions with the tooth classes during early development (See Fig. 5, Supplementary Table 2, and the discussion for a potential landmark after jaw development completed). In contrast, upper jaw is made by a fusion of the medial/lateral nasal eminences and maxillary process, which give rise to premaxilla and maxilla, respectively. Thus, the boundary of these components could be used as a landmark to correlate the tooth types, the position of jaw primordium, and the homeobox expression. It is also known that the upper jaw incisors only grow from the premaxilla, and the canine grows at the distal end of the maxilla (Fig. 5, see also Figs 1A and 4A). Accordingly, when we observed the homeobox code expression in the upper jaw primordium, we realized that the homeobox expression was highly conserved between mouse (Supplementary Fig. 1), and opossum (Fig. 1C). *Alx3* expression in the upper jaw primordium was restricted to the nasal eminences. *Msx1* expression was detected both in the nasal eminences and distal 2/3 of the maxillary process. *BarX1* was expressed in the proximal 2/3 of the maxillary process, thus there is a significant overlap of *Msx1* domain and *BarX1* domain in the middle 1/3 of the maxillary process. Therefore, the future canine region likely corresponds to the *Alx3*-negative/*Msx1*-positive/*BarX1*-negative domain (Fig. 5). This observation is consistent with our inference that the premolar region will correspond to the *Msx1*/*BarX1*-double-positive domain (Fig. 5).

### Msx1 and BarX1 expressions in ferret jaw primordium

The difference in the homeobox code expression in the mandibular arches of mouse and opossum embryos may simply reflect the difference of placentals and marsupials. We thus examined the expression of *Msx1* and *BarX1* in ferret embryos, because ferret is a placental mammal with all tooth classes (dental formula: 3131/3132, Fig. 4A). In E20 ferret embryos, in which left and right mandibular arches began to fuse at the midline, a large *Msx1*-*BarX1*-double-positive region was observed both in the maxillary process and the mandibular arch (Fig. 4B,C). We also observed the *Msx1*-positive/*BarX1*-negative region at the distal part of the maxillary process, which will likely give rise to canine (Fig. 4B,C). It is of note that the distal limit of the *BarX1* expression domain was close to the distal tip of the mandibular arch in the ferret embryos (Fig. 5), which might reflect the carnivore dentition (see also Discussion). These observations suggest that, in mammals with all tooth classes, the *Msx1*-positive/*BarX1*-negative domain and the *Msx1*/*BarX1*-double-positive domain will correspond to canine and premolar domains respectively, as indicated above (Fig. 5).

### Regulation of homeobox expression in opossum mandibular primordium

**Figure 6:**
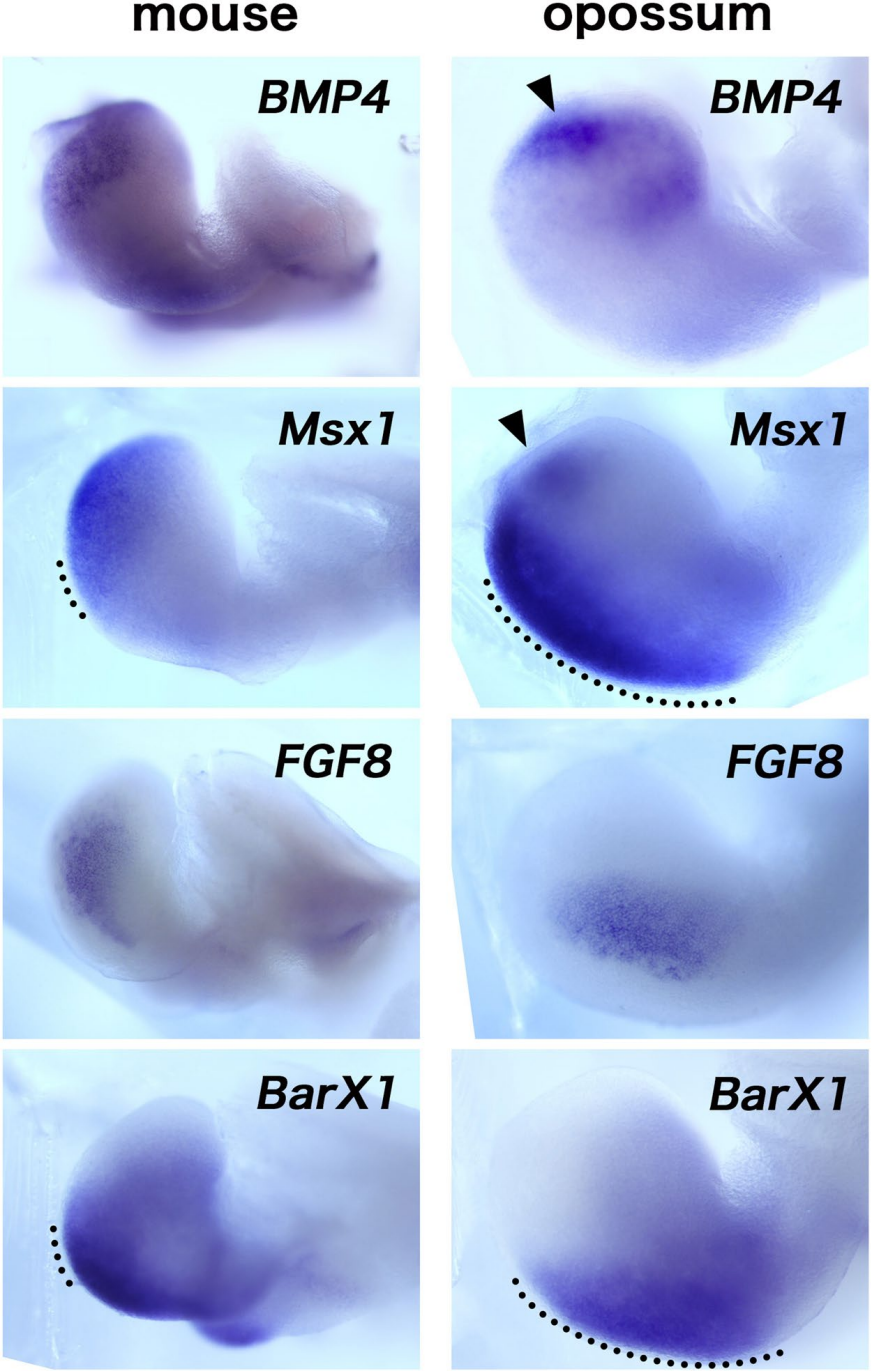
Spatial correlation of *BMP4*, *FGF8*, *Msx1*, and *BarX1* in mouse and opossum mandibular arches. Distal expression of *BMP4* and proximal expression of *FGF8* in the epithelium are well conserved in mouse and opossum. Proximal expression of *FGF8* also well coincides with that of *BarX1* in the mesenchyme of both species. While distal expression of *BMP4* coincides well with that of *Msx1* in mouse, it only overlaps with the distal part of the *Msx1* expression in opossum (arrowheads). Note **that** the *Msx1* expression domain in opossum appears to be subdivided into distal, weak region, and proximal, strong regions. Dotted lines indicate the *Msx1*/*BarX1*-double-positive domains.

It has extensively been shown that, in mouse and chicken mandibular arch, while *FGF8* expressed in the proximal mandibular arch epithelium of mouse embryos induces *BarX1*, *BMP4* expressed in the distal mandibular arch epithelium induces *Msx1* and represses *BarX1*^2,7–9,32^ (See also Supplementary Fig. 1), contributing to the reciprocal expression of *Msx1* and *BarX1*, with a small overlap (Fig. 6). If this is also the case in the opossum and ferret mandibular arch, how the large overlap of these genes could be possible? To obtain insights to answer this question, we have correlated the expressions of *BMP4* and *FGF8* with those of *Msx1* and *BarX1* in the mouse and opossum mandibular arches (Fig. 6). In the mouse mandibular arch, distal *BMP4* and proximal *FGF8* expressions well coincided with the distal *Msx1* and the proximal *BarX1* domains, respectively (Fig. 6, left panels). In fact, in the opossum mandibular arch, *BMP4* and *FGF8* were expressed in the distal and proximal epithelium, respectively (Fig. 6, right panels), very similar to those in mouse. While the *BarX1* expression domain well coincided with the *FGF8* expression in the opossum mandibular arch, however, *Msx1* expression appears to be subdivided into weak distal tip expression and stronger proximal expression, and *BMP4* expression only coincided with the distal *Msx1*–positive domain (Fig. 6, right panels). Thus, in the opossum mandibular arch, the proximal *Msx1* expression might be activated by other mechanism(s).

**Figure 7:**
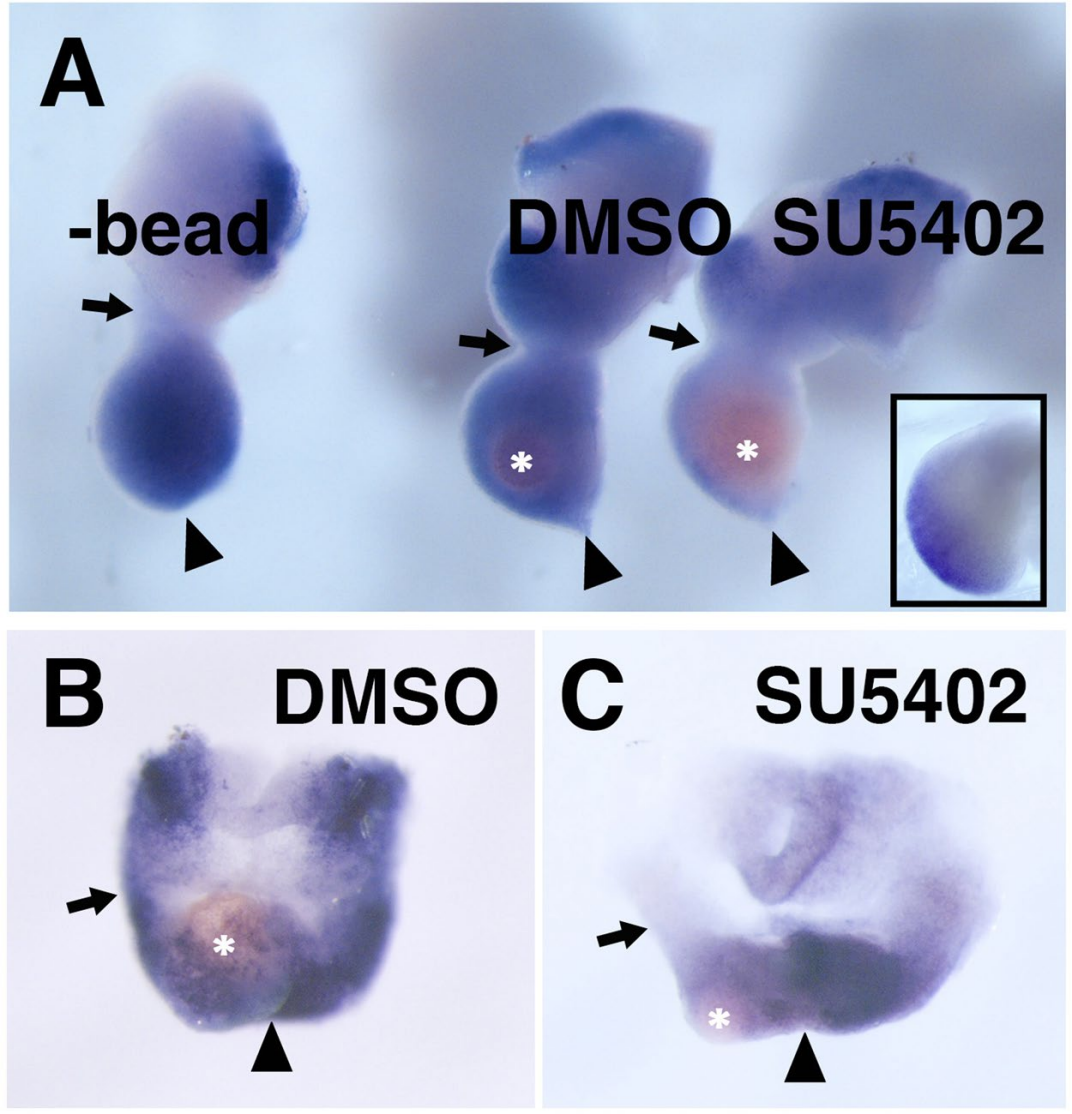
FGF signal is required for the proximal expression of *Msx1* in the mandibular arch. (**A**) Right mandibular arch explants from mouse embryos cultured for 24 hours. With DMSO-bead (n = 4/5) and without bead (n = 5/5), *Msx1* is expressed in the entire mesenchyme of the arch. In contrast, SU5402-bead-implanted explant shows a reduced *Msx1* expression (n = 5/5). Inset indicates a similar explant cultured only for 1 hour (n = 4/4). (**B**) A DMSO-bead-implanted mandibular arch of opossum embryo (n = 2/2). (**C**) The SU5402-bead-implanted right mandibular arch shows reduced *Msx1* expression nearby the bead (n = 4/4). Asterisks, arrows, and arrowheads indicate the position of implanted beads, proximal margin, and distal margin of the mandibular arches, respectively.

Since the proximal *Msx1* expression domain in opossum appeared to overlap with the *FGF8* domain (Fig. 6, right panels), and since exogenous FGF proteins could activate *Msx1* expression in mouse mandibular arch explants^7,8^ (See also Discussion section), FGF signaling could activate *Msx1* expression in the opossum jaw primordium. Interestingly, when mandibular explants were prepared from mouse and quail embryos, with no implants or control DMSO-bead implantation, *Msx1* expression could be detected all the way from distal tip to the proximal end of the explanted mandibular arch (Fig. 7A, Supplementary Fig. 3). This expanded expression of *Msx1* was repressed by an inhibition of FGF signaling with a SU5402-bead implantation. Similar results were obtained with the opossum mandibular explants (Fig. 7B,C), indicating that FGF signal is generally required for the proximal *Msx1* expression in the mandibular arch. Thus, *in vivo*, the proximal FGF-dependent *Msx1* expression in the mandibular arch should be repressed by unknown mechanism(s) in mouse or quail, but in opossum, such repression might be either weaker or only effective in the very proximal part of the arch.

## Discussion

In this study, we have compared the expression of the “homeobox code” transcription factors in the jaw primordia of mammalian species, and show that the expression pattern is well conserved in species with all 4 tooth-classes, such as opossum and ferret, but partially differs from that in mouse. The difference seems to be originated from the FGF-dependent activation of *Msx1*, leading to the broad overlap of *BarX1* and *Msx1*, which will contribute to the premolar differentiation in opossum and ferret. We also indicate that the *Alx3*-negative, *Msx1*-positive, *BarX1*-negative domain seems to correspond to the canine. Accordingly, we suggest a revised version of the homeobox code model for mammals with all tooth classes (Fig. 5). This revised homeobox code fits well with a previous observation that the mesenchyme cells of tooth germs for multi-cusp tooth express *BarX1*^16^. This code had likely been established in the prototypical heterodont mammaliaformes/mammals, or at least in common ancestors of marsupial and placental mammals.

As most mammals including carnivores like ferret are diphyodont, initial “deciduous” teeth may be replaced with consecutive “permanent” teeth except for the molars^37^. In contrast, there is no tooth replacement in mouse. Thus, it should be carefully considered whether the homeobox code in the developing jaw would be involved in the fate determination of both sets, or only the initial set of the teeth. Interestingly, only the third premolars will be replaced with permanent teeth growing from the same dental lamina, and other initial teeth will be retained in marsupials including opossum^38,39^. Because the morphology of the second set of the teeth appears similar to that of the initial set, the second tooth germ may retain the initial homeobox code expression, or the memory of the code.

### Establishment of mammalian homeobox code

Previous studies in mouse and chicken showed that the proximal-distal order of the homeobox code is largely conserved, thus the origin of the code precedes the separation of these lineages from the common ancestor. Accordingly, McCollum and Sharpe have suggested that the proximal-distal patterning of the jaw elements by differential homeobox expression established in a common ancestor of mammals and birds was thereafter co-opted for the heterodonty in the synapsid lineage^3^.

Yet, to achieve a divergent tooth patterns found in current mammals, the code would have likely been further modified. As indicated in the previous studies, and as confirmed in this study, distal *Msx1* and proximal *BarX1* domains have relatively small overlap in the mouse mandibular arch. In chicken embryos, these domains were previously reported as “complementary”^8^, like those in mouse embryos. However, when we looked at their data, we noticed a significant overlap of these domains, and we subsequently confirmed the overlap in quail embryos (Supplemental Fig. 3). Thus, as McCollum and Sharpe suggested, the prototypical homeobox code had been established in the common ancestor of mammals and birds, and it has been modified in mouse, particularly in the lower jaw. How modifications of the code might contribute to a variety of dental formula in mammalian species remain to be examined. It seems noteworthy that the distal limit of the *BarX1* expression is very close to the distal tip of mandibular arch in ferret embryo, leaving a relatively small *BarX1*-negative domain. Because ferret, like other carnivora species, has canines in the front of the jaw, we speculate that such shift of *BarX1* expression would contribute to the carnivorous adaptation.

It remains unclear how the proximal limit of the *Msx1* expression is determined. As shown above, an isolation of the mandibular arch from the embryo provokes *Msx1* expression in the entire mandibular arch taken from quail, mouse, and opossum. Thus, we hypothesize that inhibitory signal(s) would come from the rest of the embryonic body. This ectopic expression of *Msx1* in culture is inhibited by an application of FGF signal inhibitor SU5402, suggesting the requirement of the FGF signal for the proximal *Msx1* expression. In fact, some previous studies showed that, in the absence of the epithelium, exogenous FGF could induce *Msx1* in the mouse mandibular explants^7,9^. Yet, while *Msx1* expression nearby the FGF8 source is limited in the normal development, FGF signal itself likely affects more broadly, as other FGF targets, such as *BarX1*^2^ and *Lhx6*/7^40^, are expressed more proximally. Interestingly, *Msx1* activation by FGF in the chick limb bud is mediated by NF-κB^41^, suggesting that not the canonical MAP kinase pathway, but PI3 kinase/Akt or other pathway(s) could be used for the *Msx1* expression. Thus, in the proximal part of the lower jaw primordia, this non-canonical FGF signaling may activate *Msx1* expression, and this regulation may be further modified by unknown mechanism(s) at different degrees in various mammalian species. How co-expression of the *Msx1* and *BarX1* would affect the tooth morphogenesis is unknown, but a molecular interaction of Msx1 and BarX1 proteins^16^ could regulate a unique set of target genes to achieve it.

### Mouse upper VS lower tooth

Muroid rodents including mouse have a large diastema, separating remaining incisor and molars, while many rodent species such as squirrel possess one premolar adjacent to the first molar (see Supplementary Table 2)^42^. Previous mouse studies have shown that many rudimental tooth primordia are transiently formed in the future diastema of developing upper jaw, and such primordia degenerate afterwards by apoptosis^43–45^. In contrast, 2 rudimental tooth buds distally ranging the first molar is observed in the mouse lower jaw, and only epithelial thickening can be transiently recognized in the future diastema^43,44^. The difference of diastema formation, therefore, indicates that mechanisms of creating diastema in the upper and lower jaws may differ in mouse.

The mental foramen, an exit of trigeminal nerve from the dental bone, is located at the level between the first and the second premolars in human. We examined the position of the mental foramen in various mammalian species (Supplemental Table 2, See also Figs 1A and 4A). Although some species have multiple mental foramina, the major mental foramen locates at the level between the canine and the second premolar in most cases examined (Supplementary Table 2). Thus, it seems possible to use the mental foramen as a landmark of the canine-second premolar region in the dental bone, even if the given species may lack the canine and/or the premolar. In muroid rodents, in fact, the mental foramen is found close to the molars (Supplementary Table 2, See also Figs 5, 6, and Supplementary Fig. 1), suggesting that, in the mouse lower jaw, the premolar region may be largely missing, and the distal most of the diastema may simply be the socket of the rodent-specific huge incisor root. Consistently, a relatively small *Msx1*/*BarX1* double-positive region, the premolar domain suggested in this paper (Figs 5 and 6), is observed in the mouse mandibular arch. Previous studies in mouse have shown that mutations involved in Fgf, Wnt, and Hedgehog signaling pathways result in an additional tooth in front of the first molar^12,14,15^. Such “revitalization”^46^ of the rudimental tooth may be interpreted as an atavism, as extant rodents and possibly the common ancestors of muroid and extant rodents have one premolar in the same position (see also Supplemental Table 2). Nonetheless, the fact that no more premolars or canine has ever been observed in any mouse mutants further indicates that mouse mandibular arch has a potential to grow only one premolar. It will be an interesting question for the future studies if the mechanism predicted in rodents could also be applied in other mammals with premolar diastema, such as the marsupial group Diprotodontia, including wallaby and koala, as an example of convergent evolution.

### Human condition and homeobox code genes

Human genetic studies have identified familial tooth agenesis genes, such as *Wnt10a*, *Pax9*, and *Msx1*^47–52^. In *Msx1*-deficient patients, loss of 2nd premolar is the most prominent phenotype, suggesting that *Msx1* is important for the human premolar development^47,51,52^. It is unclear, however, whether such hypodonty in the *Msx1*-deficiency is caused by the altered regional identity of the jaw, or the defective tooth development, because *Msx1* is also expressed in the tooth germ at later developmental stages. To clarify such problem, we will have to manipulate *Msx1* expression in the developing jaw primordium of mammalian species with all tooth classes at specific timing.

## Supplementary information

Supplementary information

Supplementary Movie 1

Supplementary Movie 2

Supplementary Movie 3

## Data Availability

All data generated or analyzed during this study are included in this published article and its Supplementary Information.
