# Supplementary material for: Homeobox code model of heterodont tooth in mammals revised
Source: Sci Rep. 2019 Sep 6;9:12865. doi: 10.1038/s41598-019-49116-x (PMC6731288; doi:10.1038/s41598-019-49116-x)
Supplement: Supplementary file 1 — Supplementary information [file 41598_2019_49116_MOESM1_ESM.docx]

**Supplementary Information to:**

**Homeobox code model of heterodont tooth in mammals revised**

Yoshio Wakamatsu^1^*, Shiro Egawa^2^, Yukari Terashita^3^, Hiroshi Kawasaki^3^, Koji Tamura^2^, and Kunihiro Suzuki^4^

^1^ Department of Developmental Neuroscience, United Centers for Advanced Research and Translational Medicine (ART), Tohoku University Graduate School of Medicine, Sendai, Miyagi 980-8575, Japan

^2^ Department of Ecological Developmental Adaptability Life Sciences, Tohoku University Graduate School of Life Sciences, Sendai 980-8578, Japan

^3^ Department of Medical Neuroscience, Kanazawa University, Graduate School of Medicine, Kanazawa 920-8640, Japan.

^4^ Research Institute of Oral Science, Nihon University School of Dentistry at Matsudo, Chiba 271-8587, Japan

*Corresponding author:

Department of Developmental Neuroscience, United Centers for Advanced Research and Translational Medicine (ART), Tohoku University Graduate School of Medicine, Sendai, Miyagi 980-8575, Japan

Tel: 81-22-717-8203

Fax: 81-22-717-8205

Email: wakasama@med.tohoku.ac.jp

|  | forward/reverse |
| --- | --- |
| opossum *Msx1* | CHCAAACATAAGACCAACAG  GATACATGCTGTATCCCACG |
| opossum *BarX1* | CCTCCAGACGCAAAGAGTGC  GCTGTTCGCTGGTGGGAATG |
| opossum *Alx3* | AGGTCTCCAAAGTCGCCAGC  CTGGGAGACTTGTAGTCACC |
| opossum *Dlx1* | TTCTCCCATGTCCCACGGAC  ACATGAGCTGAGGTTGCTGC |
| opossum *Dlx2* | GATGGGCTCGTACCAGTACC  GGACCTCTCCGCTCTTCCAC |
| opossum *FGF8* | CCTCATTCGGAGGTACCAGC  AAGGCCATGTACCAGCCTTC |
| opossum *Shh* | CCCTGGCTATCTCGGTCATG  TCACTTAGGGACACGCTGTG |
| ferret *Msx1* | TGCGCAAGCACAAGACCAAC  GGCTGGAGGAATCAGCTAGG |
| ferret *BarX1* | GCCTGGAGTCTCCCACCAAG  TCACTGTCTTCGCAAGAGAG |
| mouse *Msx1* | TCCTTCCTAGGAAGCTCTGC  ACCTACCTTGGTGAAGCACC |

**Supplementary Table 1: List of primers for genomic/RT-PCR**

| Order | Scientific name | Species name | Specimen number | Dental formula  (lower jaw) | Position of mental foramen | Position of sub-mental foramen |
| --- | --- | --- | --- | --- | --- | --- |
| Didelphimorphia | *Monodelphis domestica* | Short-tailed gray opossum | KQN-m-011 | 4/1/3/4 | P1 - P2 | None |
| Hyracoidia | *Procavia capensis* | Rock hyracks | NMNS-1807 | 2/0/4/3 | P1 - P2 | None |
| Primates | *Macaca fuscata* | Japanese macaque | NMNS -99 | 2/1/2/3 | P2 | None |
| Primates | *Saimiri sp.* | Squirrel monkey | NMNS -100 | 2/1/3/3 | C - P1 | P1 - P2 |
| Primates | *Callithrix jacchus* | Common marmoset | NMNS -101 | 2/1/3/3 | P1 - P2 | None |
| Primates | *Mandrillus sphinx* | Mandrill | INM-1-047964 | 2/1/2/3 | P1 - P2 | P1 |
| Rodentia | *Mus musculus* | House mouse | KQN-m-032 | 1/0/0/3 | Diastema† | None |
| Rodentia | *Cavia porcellus* | Guinea pig | NMNS -169 | 1/0/1/3 | Diastema† | None |
| Rodentia | *Sciurus niger* | Fox squirrel | NMNS -176 | 1/0/1/3 | Diastema† | None |
| Rodentia | *Castor nanadensis* | Beaver | NMNS -174 | 1/0/1/3 | Diastema† | None |
| Rodentia | *Petaurista leucogenys* | Flying squirrel | NMNS -93 | 1/0/1/3 | Diastema† | None |
| Rodentia | *Erethizon dorsatum* | American porcupine | NMNS -183 | 1/0/1/3 | Diastema† | None |
| Lagomorpha | *Pentalagus furnessi* | Amami rabbit | NMNS -171 | 1/0/2/3 | Diastema† | None |
| Soricomorpha | *Mogera wogura* | Japanese mole | NMNS -122 | 3/1/4/3 | P1 | P2 |
| Cetartiodactyla | *Sus scrofa* | Japanese boar | NMNS -11 | 3/1/4/3 | P1 - P2 | P1 - P4 |
| Cetartiodactyla | *Cervus nippon* | Japanese　dear | NMNS -4 | 3/1/3/3 | Diastema  C – P1 | None |
| Cetartiodactyla | *Muntiacus reevesi* | Reeves’s muntjac | NMNS -108 | 3/1/3/3 | Diastema  C – P1 | P2 |
| Carnivora | *Canis lupus* | Wolf | INM-1-001568 | 3/1/4/3 | P2 | P2, P3 |
| Carnivora | *Canis latrans* | Coyote | INM-1-007527 | 3/1/4/3 | P1 – P2 | P3 |
| Carnivora | *Nyctereutes procyonoides* | Raccoon dog | NMNS -71 | 3/1/4/3 | P1 | P2 - P3 |
| Carnivora | *Felis concolor* | Mountain lion | INM-1-007579 | 3/1/3/0 | C – P1 | P1 |
| Carnivora | *Lynx rufus* | Bailey’s bobcat | NMNS -199 | 3/1/2/1 | C – P1 | P1 |
| Carnivora | *Ursus arctos* | Brown bear | NMNS -124 | 3/1/4/3 | P1 | P2 – P4 |
| Carnivora | *Selenarctos thibetanus japonicus* | Asiatic black bear | NMNS -50 | 3/1/4/3 | P4 | None |
| Carnivora | *Mustela putorius furo* | Ferret | SUG-m-001 | 3/1/3/2 | P1 – P2 | P3 |
| Carnivora | *Martes melampus* | Japanese marten | NMNS -79 | 3/1/4/2 | P1 | P2 - P3 |
| [Perissodactyla](https://en.wikipedia.org/wiki/Perissodactyla) | *Tapirus indicus* | Asian tapir | NMNS -130 | 3/1/3/3 | P1 | None |
| [Perissodactyla](https://en.wikipedia.org/wiki/Perissodactyla) | *Equus caballus* | Horse (female*) | NMNS -131 | 3/0/3/3 | Diastema  near P1 | None |

**Supplementary Table 2: Position of mental foramen in various mammalian species.**  Collections at Nihon University, National Museum of Nature and Science, and Ibaragi Nature Museum are indicated in the collection number by KOM or SUG, NMNS, and INM, respectively. †Mental foramen is found at the proximal end of diastema close to molar (mouse) or the only premolar they have (other rodents and rabbit). *Because male horse has canines, sex is indicated for this specimen.

**
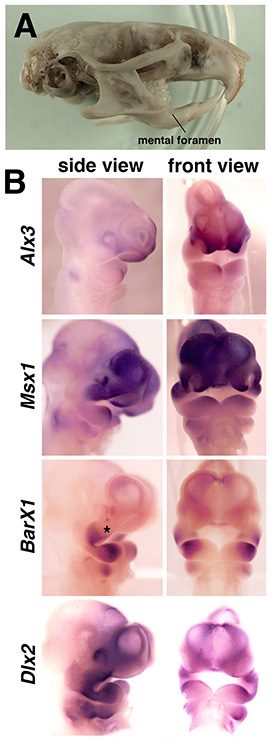
**

**Supplementary** **Fig. 1.** (A) Mouse skull, showing the position of mental foramen close to the first molar in the mandibular diastema. (B) Expression of homeobox code transcription factors in the jaw primordium of E10.5 mouse embryos. See large overlap of *Msx1* and *BarX1* both in maxillary process. Asterisk indicates *Alx3*-negative, *Msx1*-positive, *BarX1*-negative domain in the maxillary process.


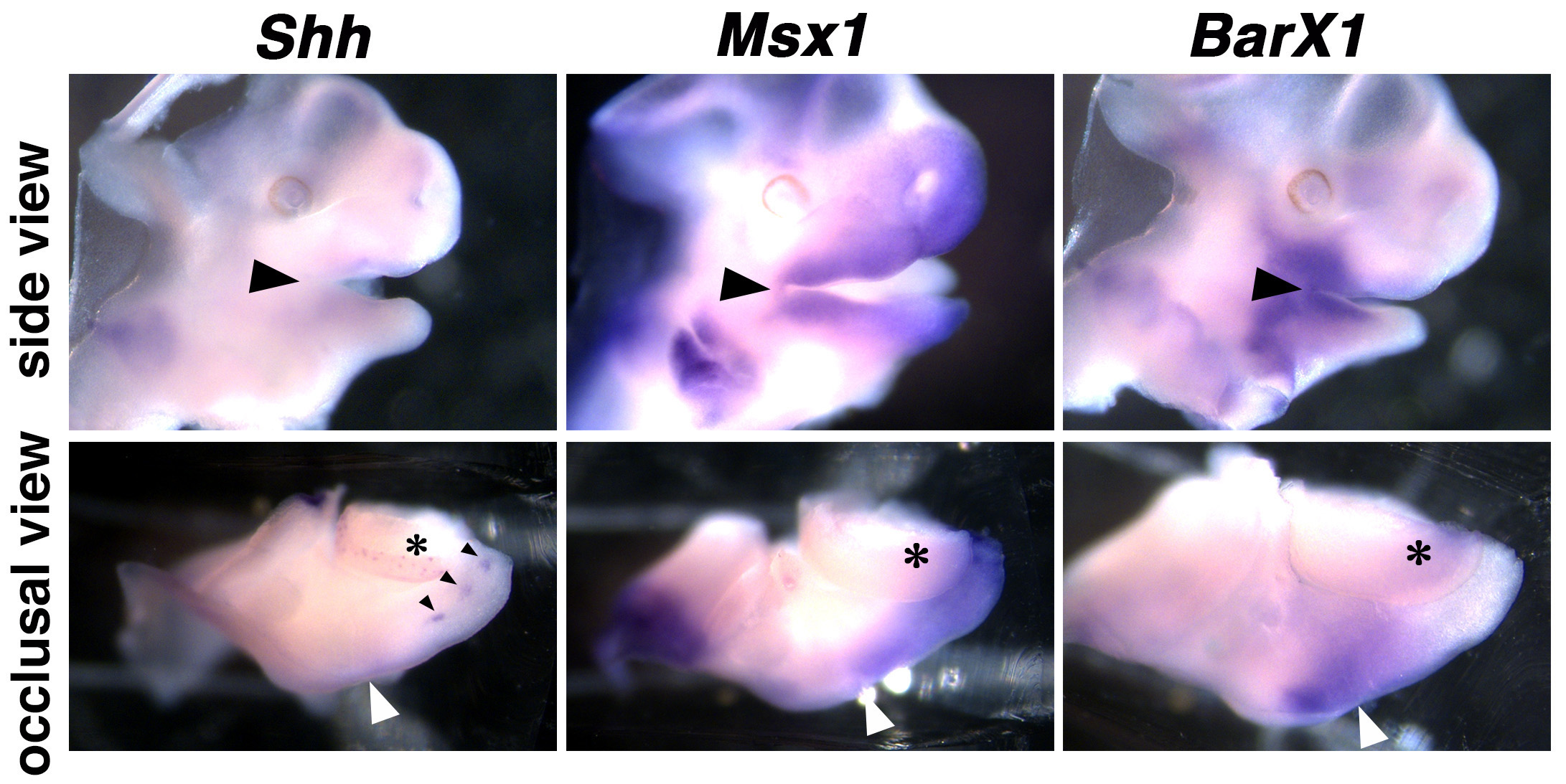


**Supplementary** **Fig. 2.** (A) Expression of *Shh*, *Msx1* and *BarX1* in E13.5 opossum embryos. All embryos were bisected at the midline. For better comparisons, pictures may have been flipped. Upper and lower rows show side and occlusal views, respectively. Large arrowheads indicate junctions of the upper and lower jaws. Small arrowheads point *Shh* expression in the dental lamina. Asterisks indicate tongues.

**
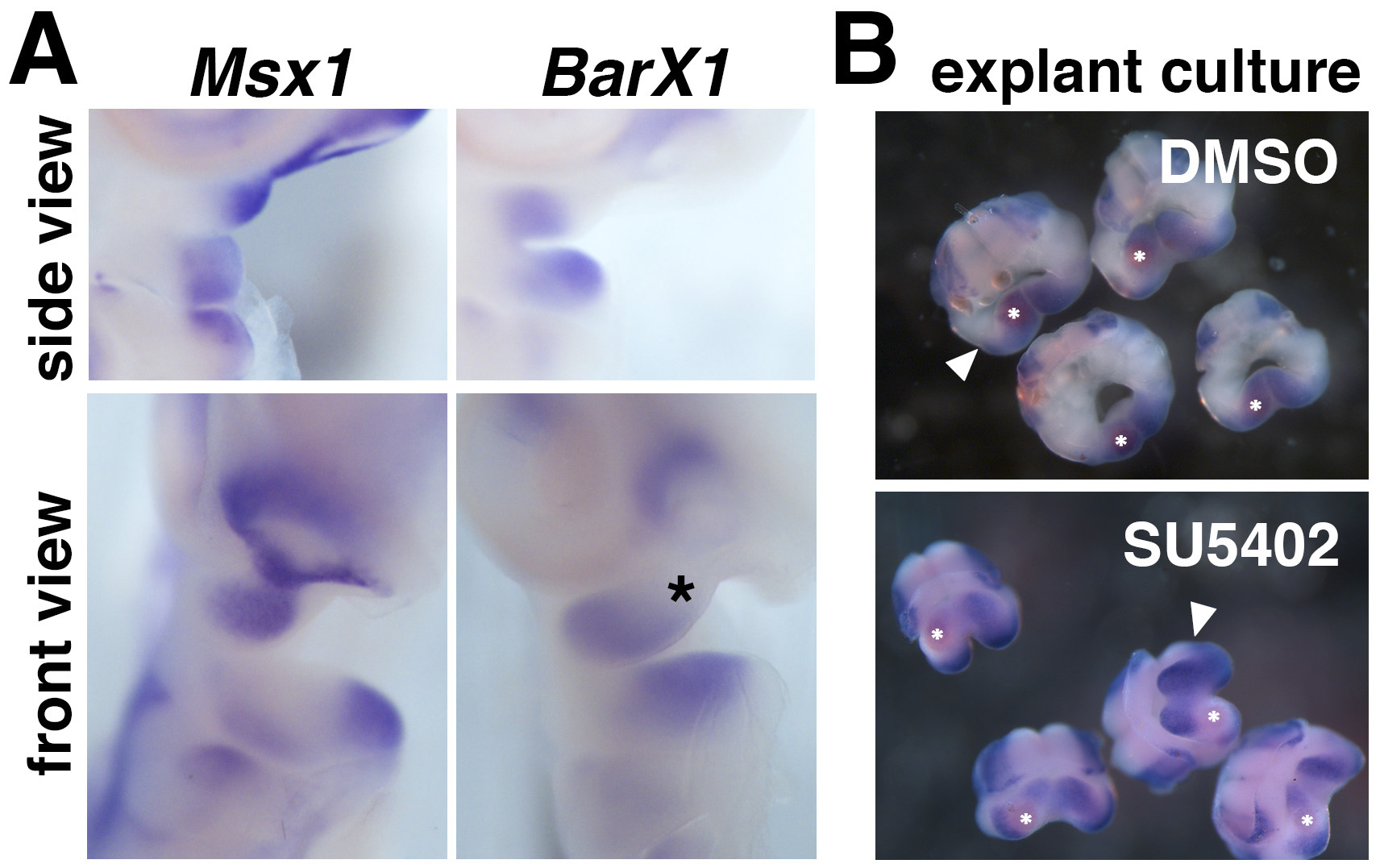
**

**Supplementary** **Fig. 3.** (A) Expression of *Msx1* and *BarX1* in E3.5 quail embryos. Asterisk indicates *Msx1*-positive, *BarX1*-negative domain in the maxillary process. (B) Quail mandibular explants either with DMSO-beads or SU5402-beads in the right arch, showing *Msx1* expression. Asterisks indicate the position of the beads. Proximal margin of the mandibular arch is indicated by arrowhead. The explants show proximal expansion of *Msx1* expression (see distally-restricted expression in vivo in A). The explants with SU5402-beads show a reduced *Msx1* expression nearby the beads (n=4/4).
